# Supplementary material for: Nuclear translocation of spike mRNA and protein is a novel pathogenic feature of SARS-CoV-2
Source: bioRxiv. 2022 Sep 27:2022.09.27.509633. Preprint. [Version 1] doi: 10.1101/2022.09.27.509633 (PMC9536038; doi:10.1101/2022.09.27.509633)
Supplement: 1 [file NIHPP2022.09.27.509633V1-supplement-1.pdf]

bioRxiv preprint doi: https://doi.org/10.1101/2022.09.27.509633; this version posted September 27, 2022. The copyright holder for this preprint (which was not certified by peer review) is the author/funder. This article is a US Government work. It is not subject to copyright under 17 USC 105 and is also made available for use under a CC0 license.

Fig S1. NLS prediction in the S protein of pathogenic coronaviruses. All categories of NLS motifs were searched in the S protein sequence using the web-based program PSORT II (https://psort.hgc.jp/form2.html) [27] for the S protein ORF amino acid sequence of SARS-CoV-2 (USA/WA-CDC-WA1/2020 isolate, GenBank accession no. MN985325) (Query 1), SARS-CoV (Urbani strain, GenBank accession no. AY278741) (Query 2), or MERS-CoV (GenBank accession no. NC_019843.3) (Query 3).

bioRxiv preprint doi: https://doi.org/10.1101/2022.09.27.509633; this version posted September 27, 2022. The copyright holder for this preprint (which was not certified by peer review) is the author/funder. This article is a US Government work. It is not subject to copyright under 17 USC 105 and is also made available for use under a CC0 license.

Fig S2. Detection of the nuclear translocation of S mRNA and **S protein.** Confocal images of SARS-CoV-2-infected airway epithelium (described in Fig 2) were analyzed for spot-to-spot colocalization using Imaris image analysis software (Oxford Instruments). The left panel shows the confocal images, the middle panel shows spot-to-spot colocalization, and the right panel shows merged confocal images and spot-to-spot colocalization. Spot-to-spot colocalization between the nucleus and S protein or S mRNA is indicated by a different color. The images represent multiple independent cross sections of the SARS-CoV-2-infected airway epithelium (from 3 independent donors).

Fig S3. The translocation of S mRNA and S protein includes both the inside and outer surface of the nucleus. From the images shown in Fig 3, the signals of S mRNA and S protein were plotted in the graph by Imaris image analysis software. The distance and intensity of all S mRNA or S protein from the nuclear surface (considered 0) were plotted. A negative value indicates that S mRNA or S protein resides inside the nucleus. The higher the negative value is, the farther the distance from the nuclear surface. In contrast, a positive value indicates that S mRNA or S protein resides on the nucleus surface and beyond in the cytoplasm. The higher the positive value is, the farther the distance from the nuclear surface.

Fig S4. The SARS-CoV S protein does not translocate into the nucleus. A four-week pseudostratified airway epithelium was infected with SARS-CoV at an MOI of 0.1 for four days, fixed, paraffin-embedded and sectioned at a thickness of 5 μm for immunohistochemistry slide preparation. S protein (shown in cyan) was detected by immunofluorescence-based protein

bioRxiv preprint doi: https://doi.org/10.1101/2022.09.27.509633; this version posted September 27, 2022. The copyright holder for this preprint (which was not certified by peer review) is the author/funder. This article is a US Government work. It is not subject to copyright under 17 USC 105 and is also made available for use under a CC0 license.

detection using SARS-CoV/SARS-CoV-2 S specific rabbit polyclonal primary antibody and anti-rabbit secondary antibody. The confocal image was analyzed for spot-to-spot colocalization using Imaris image analysis software. The left panel shows a confocal image, the middle panel shows spot-to-spot colocalization, and the right panel shows merged confocal images and spot-to-spot colocalization. The images represent multiple independent cross sections of SARS-CoV-infected airway epithelium (at least two donors).

Fig S5. S mRNA and S protein colocalization was spatially evident in all possible ways inside the infected cell. The confocal images shown in Figs. 3 & 4 were further visualized at a higher magnification to detect S mRNA and S protein colocalization spatially. S protein and S mRNA distribution and colocalization in the cytoplasm (top panel), on the nuclear surface (middle panel) and inside the nucleus (bottom panel). The specific region of colocalization is indicated by a white spot. The colors were made translucent to show colocalization. Scale bar 0.2 μm.

Fig S6. NLS motif prediction in the N protein of pathogenic coronaviruses. All categories of NLS motifs were searched in the N protein sequence using the web-based program PSORT II (https://psort.hgc.jp/form2.html) [27] for the N protein ORF of SARS-CoV-2 (USA/WA-CDC-WA1/2020 isolate, GenBank accession no. MN985325) (Query 1), SARS-CoV (Urbani strain, GenBank accession no. AY278741) (Query 2), or MERS-CoV (GenBank accession no. NC_019843.3) (Query 3).

bioRxiv preprint doi: https://doi.org/10.1101/2022.09.27.509633; this version posted September 27, 2022. The copyright holder for this preprint (which was not certified by peer review) is the author/funder. This article is a US Government work. It is not subject to copyright under 17 USC 105 and is also made available for use under a CC0 license.

Fig S7. Nuclear translocation of the N protein of pathogenic coronaviruses. Four-week pseudostratified airway epithelium was infected with SARS-CoV-2, SARS-CoV, or MERS-CoV at an MOI of 0.1 for four days, fixed, paraffin-embedded and sectioned at a thickness of 5 μm for immunohistochemistry slide preparation. SARS-CoV-2 or SARS-CoV N protein (green) was detected by a SARS-CoV/SARS-CoV-2 N protein-specific antibody. Similarly, the MERS N protein (green) was detected by the MERS N protein-specific antibody. The nucleus (shown in blue) was detected by DAPI staining. The confocal images were analyzed for spot-to-spot colocalization. The left panel shows a confocal image, the middle panel shows spot-to-spot colocalization, and the right panel shows merged confocal images and spot-to-spot colocalization. Spot colocalization between the nucleus and N protein is indicated by a different color. The images represent multiple independent technical replicates from at least one independent experiment for one donor (donor #1).

Fig S8. NLS motif distribution in the N protein in different pathogenic coronaviruses. The sequences of the N protein of the SARS-CoV-2 N protein (nCoV-WA1-2020, GenBank accession no. MN985325), SARS-CoV N protein (Urbani Strain, GenBank accession no. AY278741), and MERS-CoV N protein (HCoV-EMC/2012, GenBank accession no. NC_019843) by NCBI's constraint-based multiple alignment tool COBALT [25]. All categories of NLS motifs are shown in the colored rectangle box: pat4: green; pat7: blue; bipartite 1: black; bipartite 2: orange.

S1 dataset 1. Prediction of SARS-CoV-2 S protein and genome interaction

S2 dataset 2. Prediction of SARS-CoV-2 N protein and genome interaction
